# Translation, data quality, reliability, validity and responsiveness of the Norwegian version of the Effective Musculoskeletal Consumer Scale (EC-17)

**DOI:** 10.1186/1471-2474-11-21

**Published:** 2010-01-29

**Authors:** Bente Hamnes, Andrew Garratt, Ingvild Kjeken, Elizabeth Kristjansson, Kåre B Hagen

**Affiliations:** 1 Hospital for Rheumatic Diseases, M. Grundvigsvei 6, 2609 Lillehammer, Norway; 2 National Resource Centre for Rehabilitation in Rheumatology, Diakonhjemmet Hospital, Boks 23, Vinderen, 0319 Oslo, Norway; 3 School of Psychology, Faculty of Social Sciences, University of Ottawa, 1 Stewart St, Ottawa, Ontario K1N 6N5, Canada

## Abstract

**Background:**

The Effective Musculoskeletal Consumer Scale (EC-17) is a self-administered questionnaire for evaluating self-management interventions that empower and educate people with rheumatic conditions. The aim of the study was to translate and evaluate the Norwegian version of EC-17 against the necessary criteria for a patient-reported outcome measure, including responsiveness to change.

**Methods:**

Data quality, reliability, validity and responsiveness were assessed in two groups. One group comprising 103 patients received a questionnaire before and at the end of a self-management programme. The second group comprising 96 patients' received the questionnaire two weeks before and on arrival of the program. Internal consistency and test-retest reliability were assessed. Construct validity was assessed through comparisons with the Brief Approach/Avoidance Coping Questionnaire, (BACQ), the Emotional Approach Coping Scale (EAC) and the General Health Questionnaire (GHQ-20). Responsiveness was assessed with the Standardised Response Mean (SRM).

**Results:**

Respondents included 66 (64%) and 52 (54%) patients from the first and second groups respectively. Levels of missing data were low for all items. There was good evidence for unidimensionality, item-total correlations ranged from 0.59 to 0.82 and Cronbach's Alpha and test-retest correlations were over 0.90. As hypothesised EC-17 scores had statistically significant low to moderate correlations with the BACQ, EAC and GHQ-20 in the range 0.26 to 0.42. Following the self-management program, EC-17 scores showed a significant improvement with an SRM of 0.48.

**Conclusion:**

The Norwegian version of the EC-17 has evidence for data quality, internal consistency and test-retest reliability, construct validity and responsiveness to change. The EC-17 seems promising as an outcome measure for evaluating self-management interventions for people with rheumatic conditions, but further studies are needed.

## Background

Self-management programs are increasingly used as a means to empower, educate and inform patients with chronic rheumatic diseases. Such interventions are designed to encourage patients to be more active and take responsibility for their own health care with aims of increased self-efficacy, coping with stress, problem solving and interactions with healthcare professionals [1,2]. Self-management programs have some evidence for effectiveness [3-9]. Evaluation has, however, been hindered by a lack of appropriate outcome measures [10-12].

One-week self-management programs addressing the needs of patients with different rheumatic diseases have been developed at the Hospital for Rheumatic Diseases in Lillehammer, Norway. Several patient-reported outcome measures (PROMs) have been used to evaluate these programs, including measures of different aspects of health status and quality of life [1,4,13,14]. Such outcomes are important for assessing long term benefits but may not be responsive to important changes in the shorter to medium term relating to patient skills in managing and taking an active role in healthcare. Moreover, a systematic review found that over 16 different outcome measures had been used to evaluate self-management interventions [4]. This has important implications for generalisability [15]. Another overview of evaluation of psychoeducational/self-management interventions presented more than 30 different outcomes from 24 studies [1,16], which indicate the complexity of the self-management education. Different interventions, often delivered in variety of environments and with different outcomes make it difficult to compare results from one study/intervention with another.

The OMERACT (Outcome Measures in Rheumatology) Effective Musculoskeletal Consumer Project was designed to address the need for appropriate outcome measures for evaluating and comparing self-management programs [15,17]. Previous research demonstrated that existing outcome measures failed to assess a number of important skills, including the ability to find and assess information, decision making and implementation, and to take part in the health care system and society [15,17]. The Effective Musculoskeletal Consumer Scale (EC-17) was developed to address the need for an instrument that assesses skills and attributes of patients as effective consumers who manage their healthcare, which is an important part of self-management. It is intended for use both to discriminate between patients with different levels of skills and for assessing the outcomes of interventions designed to improve skills. The English language version of the EC-17 was found to be acceptable to patients and have high internal consistency. The aim of this study was to translate the English language version of EC-17 [18] into Norwegian and assesses the data quality, internal consistency and test-retest reliability, construct validity and responsiveness to change of the measure.

## Methods

### Data collection

Two groups of patients with rheumatic diagnoses, aged 25-85, participated in a one-week in- patient self-management program at the Hospital for Rheumatic Diseases in Lillehammer, Norway. The recruitment of patients took place from January to April 2007. Patients in both samples were diagnosed and referred by rheumatologists and general practitioners from across Norway. The patients, mostly females and with a disease duration of over one year, received the invitation to participate in the study together with a letter with the date of their hospital stay. For purposes of assessing internal consistency, validity and responsiveness to change, 103 patients were asked to complete the EC-17 before and at the end of the one-week self-management program. For purposes of assessing test-retest reliability, 96 patients were sent a postal version of the EC-17 two weeks before attending the program and also completed the questionnaire on arrival and at the end of the one-week self-management program.

### Intervention

The one-week self-management program summarised in Table 1, is designed to help people manage their rheumatic disease and challenges in daily life. The program has the same core concepts as outpatient programmes and comprises information, discussions on how to cope with the disease and daily life, cognitive management skills, exercise, engagement in self-care and interactions with healthcare professionals [2,4,19,20]. This program is unique in that patients are hospitalized for the one week program rather than participating in a series of short modules that run over a long period of time. During the program, the inpatient education unit takes up to 16 patients and 5 relatives per week within one diagnostic group. All patients receive a core program but because patients with different diagnoses have different needs, some programs have supplements. For example, patients with ankylosing spondylitis have more physical exercises, whereas fibromyalgia patients have more process-orientated therapy related to stress management and communication. Each educational session lasts for 1.5-2.5 hours and the physical activity sessions for 0.5-1 hour. In the evening group sessions, the patients are divided into small groups of approximately five participants. Relatives have their own group. They talk together for one hour under leadership of one health professional who is educated in coaching. The focus is on coping with the disease and daily life. Exercise facilities including a swimming pool, are available in the evening for individual training.

**Table 1:** The Self-Management Program

Sunday	Monday	Tuesday	Wednesday	Thursday	Friday
	Living with chronic disease (Nurse)	Coping with daily activities (Occupational Therapist)	Self-management (Nurse)	Health and social welfare (Social Worker)	Healthy eating (Dietician)
Arrival in the evening	Physical activity Swimming-pool (Physiotherapist)		Physical activity - Nordic walking	Physical activity theory and exercises (Physiotherapist)	Evaluation and end of program (Nurse)
Information regarding the coming week	Consultation rheumatologist	Rheumatic disease and treatment (Rheumatologist)	Creative activity (Occupational Therapist)		
	Group session	Group session		Group session	

Patients gave informed consent after receiving written information about the project. The regional committee for medical research ethics in Health Region East, Norway and The Norwegian Data Inspectorate approved the study.

### Instruments

The EC-17 (Table 2) is a self-administered instrument that was developed following a literature review, interviews with patients and physicians, feedback from OMERACT members and pretesting with patients [15,17]. The resulting questionnaire comprised 64 items which was reduced to 17 items on the basis of expert opinion and item testing [15]. The 17 items relate to knowledge, attitudes and behaviours about self-management skills with a five-point adjectival scale from 'never' to 'always'. The results of principal component analysis support the unidimensionality of the EC-17 [15]. The items are summed and converted to produce a score from 0 to 100 where 100 is the best possible score.

**Table 2:** EC-17 item ^a ^means (sd), frequencies, component loadings and item-total correlation (n = 116)

Scale/Item	Mean (sd)	Never	Rarely	Sometimes	Usually	Always	Component loading^b^	Item-total correlation
*EC-17 total scores *^c^:	61.27 (16.88)							
1. I know who can help me judge the quality of the information I receive about my disease	2.41 (1.00)	9.5	44.8	26.7	14.7	4.3	0.69	0.65
2. I understand the information I receive about my disease	2.85 (0.75)	12.1	68.1	15.5	1.7	2.6	0.66	0.62
3. I know how to adapt general health information to my own situation	2.47 (0.84)	4.3	54.3	28.4	10.3	2.6	0.78	0.75
4. I can be clear about what is important in my life when I make decisions about my disease	2.57 (0.85)	8.6	52.6	27.6	9.5	1.7	0.74	0.69
5. I can weigh the pros and cons of a decision about my disease	2.67 (0.68)	6.9	57.8	31.9	2.6	0.9	0.68	0.63
6. I can set realistic goals about the management of my disease	2.47 (0.80)	3.4	52.6	33.6	7.8	2.6	0.67	0.62
7. I can express my concerns well to health care providers	2.65 (0.98)	16.4	48.3	22.4	9.5	3.4	0.77	0.74
8. I know how to ask good questions about my health and my disease	2.36 (0.95)	4.3	52.6	21.6	18.1	3.4	0.85	0.82
9. I have built an open and trusting relationship, based on mutual respect, with my health care providers	2.80 (1.01)	22.4	50.9	15.5	6.9	4.3	0.72	0.67
10. I am able to play the role I want to in my health care team	2.52 (0.96)	10.3	50.0	24.1	12.1	3.4	0.64	0.60
11. I know who to work with to meet my health needs	2.46 (0.98)	8.6	50.9	22.4	13.8	4.3	0.82	0.79
12. I can be assertive to get what I need to meet my health needs	2.02 (1.09)	6.9	32.8	26.7	25.9	8.6	0.73	0.69
13. I feel a sense of control over my disease	2.22 (0.92)	3.4	40.5	36.2	14.7	5.2	0.64	0.59
14. I feel confident in making decisions about my health	2.45 (0.91)	8.6	44.0	34.5	9.5	3.4	0.80	0.76
15. I can negotiate with others about what we need to do to manage my disease	2.28 (0.93)	2.6	46.6	32.8	12.1	6.0	0.78	0.74
16. I can negotiate with the health care system about what to do to manage my disease	2.15 (1.02)	5.2	39.7	25.0	25.0	5.2	0.80	0.76
17. I can organize my life to act on decisions about how to manage my disease	2.33 (0.95)	6.0	44.0	31.0	14.7	4.3	0.71	0.66

^a ^EC-17 items use a five point scale, ranging from 0 to 4, were 0 is never and 4 is always.

^b ^The two EC-17 components derived from principal component analysis had eigenvalues of 9.21 and 1.36.

^c ^EC-17 total score is calculated by adding up the scores and converting to a scale from 0 to 100 where 100 is the best possible score.

In the present study, the EC-17 translation followed the forward and backwards procedure [21]. Two independent bilingual translators including a health professional whose first language was Norwegian, translated the original version of the EC-17 into Norwegian and had a consensus meeting. Two independent bilingual translators including a health professional whose first language was English, then back translated the Norwegian version and had a consensus meeting. The translators, two researchers and BH discussed the forward and back translation, resolving discrepancies through consensus to achieve conceptual equivalence between the Norwegian and original English version of the EC-17.

The Norwegian version of the EC-17 was pre-tested with ten inpatients with rheumatic diseases recruited from patients in the hospitals rheumatology unit. All patients were Norwegian speakers and had been diagnosed with rheumatoid arthritis, psoriatic arthritis or ankylosing spondylitis. BH interviewed the patients following self-completion, asking them to provide feedback on the questionnaire, including difficulty understanding the questions.

All patients said that the questionnaire was comprehensible and no changes were necessary.

As part of testing for construct validity, the EC-17 was compared to three instruments that assess coping and psychological status. The 12-item Brief Approach/Avoidance Coping Questionnaire (BACQ) is designed to assess approach versus avoidance coping and comprises two scales of approach and avoidance that cover cognitive, emotional and action-related domains [22]. The Norwegian version of the instrument has good evidence for reliability and validity [22]. Items use a five-point scale from disagree completely to agree completely and produce two sum scores that range from 6 to 30 where 30 is the best possible score.

The 16-item Emotional Approach Coping Scale (EAC) is designed to assess coping through emotional approach. It comprises two scales: emotional processing, which assesses active attempts to know and understand one's emotions, and emotional expression, which assesses active verbal and nonverbal attempts to communicate or symbolize one's emotional experience [23,24]. The Norwegian version of EAC is an acceptable and valid instrument for measuring emotional processing and expression in patients with rheumatic diseases [25]. Items use a four-point scale from "I usually don't do this at all" to "I usually do this a lot" and produce two mean scores from 8 to 32 where 32 is the best possible score.

The General Health Questionnaire (GHQ-20) is a 20-item screening instrument for detecting psychiatric disorders, but is also used for measuring changes in psychosocial status and psychological distress in chronic diseases [26-30]. The instrument has evidence for reliability and validity [26,31-33] and evidence for high sensitivity and specificity in Norwegian trauma patients [26,32]. Items use a four-point scale from no distress to severe distress and sum to produce a total score from 0 to 60 where 0 is the best possible score indicating no distress.

### Statistical analysis

The EC-17 was assessed for missing data at the item and scale level. Following the instrument developers, principal component analysis (PCA) [34] was used to assess the unidimensionality of the instrument after assessing the ratio of the first to second eigenvalues [15,34]. The ratios of 3:1 or higher were considered evidence for unidimensionality [34]. Internal consistency was assessed by item-total correlation and Cronbach's Alpha at the item and scale level respectively. Following the findings of the instrument developers it was expected that the 17 items would have levels of correlation of over 0.4 with the remainder of the instrument. The developers reported a Cronbach's Alpha of 0.96 for the longer-form scale and Alpha and test-retest intra-class correlation coefficients of 0.9 and above meet the more stringent reliability criterion for an outcome measure [35].

The construct validity of the EC-17 was evaluated through comparisons with scores for the other three instruments and patient characteristics. Pearson's and Spearman's rank correlation were used for continuous and categorical scales respectively. The association was interpreted as being high, moderate and weak when the correlation was over 0.60, between 0.30 and 0.60 and 0.30 or less, respectively [36]. It was hypothesised that EC-17 scores would be moderately positively correlated with BACQ Approach and negatively correlated with BACQ Avoidance, moderately negatively correlated with the GHQ-20 and moderately positively correlated with EAC Expression and Processing. It was also hypothesised that younger and more highly educated patients would have higher EC-17 scores than older less well educated patients resulting in weak positive correlations with these two variables [37,38].

The responsiveness of the EC-17 was assessed by calculating change scores for patients who had undertaken the self-management program. The standardised response mean (SRM) was calculated by dividing the mean change in EC-17 scores over the one week period by the standard deviation of the change scores. SRMs of over 0.80, 0.40-0.80 and less than 0.40 were considered high, moderate and small respectively [39,40].

SPSS for Windows (version 15.0) was used for statistical analysis.

## Results

A total of 66 (64%) patients took part in the responsiveness testing and completed both questionnaires while 52 (54%) patients took part in the test-retest. There was no drop-out among the 118 patients who accepted to participate in the study, but two were excluded in the analyses because of too many missing items. Demographic data and characteristics of the patients who underwent the self-management program are shown in Table 3. The majority of patients were females and age and disease duration were similar for both samples. Patients with fibromyalgia and Sjogrens syndrome were represented in both samples. There were no significant differences between responders and non-responders regarding age, sex and diagnosis for the two groups.

**Table 3:** Mean (sd) patient characteristics

	Test-retest		Pre-post test	
	Respondents (n = 52)	Non-respondents (n = 37)	Respondents (n = 66)	Non-respondents (n = 37)
Females n	51 (98%)	36 (97%)	60 (91%)	33 (89%)
Age, yrs	52.8 (13.0)	54.6 (9.6)	53.3 (12.1)	55.2 (12.7)
Disease duration, yrs,	6.6 (7.0)		7.4 (6,8)	
Living alone	30 (58%)		17 (26%)	
Education (<12 yrs)	29 (56%)		46 (70%)	
*Diagnoses:*				
Fibromyalgia	17 (33%)	14 (38%)	21 (32%)	7 (19%)
Sjogrens syndrome	17 (33%)	10 (27%)	7 (11%)	12 (32%)
Rheumatoid arthritis	9 (17%)	7 (19%)		
Systemic Lupus Erythematosus	9 (17%)	6 (16%)		
Osteoarthritis			22 (33%)	11 (30%)
Ankylosing spondylitis			10 (15%)	4 (11%)
Scleroderma			6 (9%)	3 (8%)

EC-17 mean item scores at the start of the self-management program ranged from 2.02 for item 12 "I can be assertive to get what I need to meet my health needs" to 2.85 for item 2 "I understand the information I receive about my disease" (Table 2). The levels of missing data were low for all items.

PCA of item responses for all patients gave a two component solution that explained 62.16% of the total variation. The first component had an eigenvalue 9.21, that was 6.76 times larger than the second component of 1.36, which is adequate evidence for unidimensionality [34]. Table 2 shows that the item-total correlations ranged from 0.59 to 0.82 for the items "I feel a sense of control over my disease" and "I know how to ask good questions about my health and my disease", respectively. Cronbach's Alpha for the 17-item scale was 0.95. The test-retest intraclass correlation coefficient was 0.90.

The results of testing for construct validity are shown in Table 4. The correlation with the BACQ Approach scale was significant and of a small to moderate level, while there was no correlation with BACQ Avoidance. The correlations with the EAC were of a lower level but statistically significant. The correlation with the GHQ-20 were also low but significant. There were no statistically significant correlations between EC-17 and age or education.

**Table 4:** Pearson correlation coefficients between EC-17 scores and BACQ, EAC, GHQ-20 (n = 66), age and education (n = 116)

Instrument	Correlation	P value
The Brief Approach/Avoidance Coping Questionnaire:		
Approach	0.42	0.01
Avoidance	- 0.07	0.57
Emotional Approach Coping Scale:		
Processing	0.34	0.01
Expression	0.42	0.01
General Health Questionnaire-20	-0.26	0.05
Age	0.11	0.23
Education	-0.00	0.98

There was a significant improvement in EC-17 scores following the self-management program of 4.39 (sd = 9.25) on the 0-100 scale where 100 is the best possible scale (Table 5). The SRM was 0.48 which was larger than SRMs for all but the GHQ-20 which produced an SRM of 0.75.

**Table 5:** Mean (SD) scores and responsiveness of the EC-17, BACQ, EAC and GHQ-20 (n = 66)

Instrument	Pre-test	Post-test	Change score	SRM^a^
EC-17 ^b^	62.55 (15.34)	66.93 (13.37)**	4.39 (9.25)	0.48
EAC processing ^c^	2.83 (0.66)	2.93 (0.66)	0.10 (0.52)	0.19
EAC expression	2.76 (0.63)	2.82 (0.57)	0.06 (0.45)	0.13
BACQ Approach^d^	3.42 (0.54)	3.54 (0.55)	0.12 (0.46)	0.25
BACQ Avoidance	3.24 (1.04)	3.10 (0.56)	-0.14 (0.93)	0.15
GHQ-20^e^	22.94 (9.96)	17.55 (8.89)**	-5.39 (7.15)	0.75

^a ^SRM standardised response mean = mean change in score divided by the standard deviation of the change in scores.

^b ^EC-17 is scored from 0 to 100 where 100 is the best possible score.

^c ^The EAC is scored from 1 to 4 where 4 is the best possible score.

^d ^The BACQ is scored from 1 to 5 where 5 is the best possible score.

^e ^GHQ-20 is scored from 0 to 60 where 0 is the best possible score.

Asterisks denote statistical significance: *p < 0.05; **p < 0.01.

## Discussion

In this, study, we assessed the data quality, reliability, validity and responsiveness of the Norwegian version of the EC-17. Following forward backwards translation, the Norwegian version was clearly understood by patients involved in pre-testing the questionnaire. The 17-items of the Norwegian EC-17 questionnaire had low levels of missing data for the two groups of patients who took part in the assessment of responsiveness and test-retest, which is further evidence for the acceptability of the instrument.

The results of PCA supported the unidimensionality of the EC-17. Both internal consistency and test-retest reliability estimates met widely accepted standards for the use of such instruments. The coefficients also met the more stringent criterion for use in individual patients. The results of validity testing generally followed the a priori hypotheses with EC-17 scores having low to moderate correlations with the other questionnaires and variables. However, there were no statistically significant correlations with BACQ Avoidance, age and education.

The EC-17 was found to have evidence for responsiveness in this group of patients, however, responsiveness should be further assessed at longer-term follow up and in relation to other interventions that are designed to improve self-management. It is also recommended that other outcome measures are included alongside the EC-17 including disease specific and general measures of quality of life. This would promote further understanding of the relationship between the skills and attributes of patients as effective consumers and health outcomes more generally.

The results of responsiveness testing for the EC-17 were, however, encouraging given the short one-week interval of the study. From a self-management perspective, learning is a process, and to change attitudes and put new skills in to practice usually takes more than a week. There are three components of educational objectives which form a hierarchy - affective, cognitive and psychomotor - meaning that learning at the higher levels is dependent on having attained prerequisite knowledge and skills at lower levels [41]. Several items within the EC-17 require high levels of knowledge, attitudes and skills. The items 5 and 16 relating to weighing up the cost and benefits of decisions relating to ones disease and to negotiate with the health care system about what to do to manage ones disease, are examples of skills at high levels. It may also be that the self-management program was not as effective as intended and hence large changes in the EC-17 could not be expected.

The response rate for the patients responding before and after the management program was satisfactory at 64%, however the response rate of 54% for the test-retest group was rather low. The comparison of respondents and non-respondents showed, however, that there were no significant differences in age, sex and diagnoses. However, non-respondents were somewhat older in both groups, indicating that selection bias can not be ruled out.

The results of testing for construct validity were overall supportive of the hypotheses, however, the low correlations between EC-17 scores and age and education were insignificant. The correlation with age was even positive, which was contrary to expectations. The inclusion of very few younger patients who are expected to be more consumer-minded, may have contributed to this finding. Items within the EC-17 may not wholly reflect consumer skills in practice but rather the subjective perceptions of patients. Patient responses may be dependent on their expectations as consumers and users of information. Finally, the development of the EC-17 which involved collaboration between people with chronic rheumatic disease and health professionals [17], where the participants gave feedback regarding relevance, form and language, may have ensured that items were of equal meaning and relevance to all groups of patients thereby negating differential item functioning across groups. These issues should be considered alongside future evaluation and testing of the EC-17.

The original EC-17 which was developed and tested with patients from Australia and Canada [15], is currently being used to evaluate a program for RA in Ireland and will in the near future be used in New Zealand [15]. Our study provides the first evidence for a translated version of the EC-17 and we have demonstrated that the instrument performs satisfactorily and comparably with the original version. Furthermore, our study provides additional evidence for construct validity. Further translations of the EC-17 with accompanying evaluations will serve to broaden the outcome measures available for evaluating interventions and programs designed to enhance patient skills. Moreover, there will be greater scope for international collaboration and meta-analyses if a common primary outcome measure such as the EC-17 is agreed upon.

Patient education is described as one out of four main responsibilities of the specialist health care within Norway [42]. Most of the hospitals have established patient education units where self-management programs are widely implemented for groups of patients with chronic diseases. Today, few instruments are available to measure effectiveness of such programs. The EC-17 is especially relevant for measuring effects of the self-management programme described here, with instrument content mirroring the goals of the programme. As such the EC-17 provides important information for both clinicians and policymakers within health care.

## Conclusions

The EC-17 shows great promise as a measure of the effectiveness of self-management programmes in patients with rheumatic diseases. The questionnaire was well understood by the patients and easy to complete. The instrument has evidence for data quality, internal consistency and test-retest reliability and validity. Preliminary evidence was also found for the responsiveness of the EC-17, the instrument showing significant change in patients completing a one week self-management programme. However, further evaluative studies of the EC-17 in different clinical settings are needed.

## Competing interests

The authors declare that they have no competing interests.

## Authors' contributions

BH, KBH and IK conceived the study, EK developed the original instrument EC-17; BH collected the data; KBH, AG IK and BH performed the statistical analyses; BH, KBH, IK and AG drafted the manuscript. All authors read and approved the final manuscript.

## Pre-publication history

The pre-publication history for this paper can be accessed here:

http://www.biomedcentral.com/1471-2474/11/21/prepub

## References

[B1] Mulligan K Newman SP Taal E Hazes M Rasker JJ The design and evaluation of psychoeducational/self-management interventions J Rheumatol 2005 32 2470 2474 16331789

[B2] Newman S Mulligan K Steed L What is meant by self-management and how can its efficacy be established? Rheumatology (Oxford) 2001 40 1 4 10.1093/rheumatology/40.1.1 11157134

[B3] Astin JA Beckner W Soeken K Hochberg MC Berman B Psychological interventions for rheumatoid arthritis: a meta-analysis of randomized controlled trials Arthritis Rheum 2002 47 291 302 10.1002/art.10416 12115160

[B4] Newman S Steed L Mulligan K Self-management interventions for chronic illness Lancet 2004 364 1523 1537 10.1016/S0140-6736(04)17277-2 15500899

[B5] Niedermann K Fransen J Knols R Uebelhart D Gap between short- and long-term effects of patient education in rheumatoid arthritis patients: a systematic review Arthritis Rheum 2004 51 388 398 10.1002/art.20399 15188324

[B6] Savelkoul M de WL Post M Stimulating active coping in patients with rheumatic diseases: a systematic review of controlled group intervention studies Patient Educ Couns 2003 50 133 143 12781928 10.1016/s0738-3991(02)00121-0

[B7] Warsi A LaValley MP Wang PS Avorn J Solomon DH Arthritis self-management education programs: a meta-analysis of the effect on pain and disability Arthritis Rheum 2003 48 2207 2213 10.1002/art.11210 12905474

[B8] Warsi A Wang PS LaValley MP Avorn J Solomon DH Self-management education programs in chronic disease: a systematic review and methodological critique of the literature Arch Intern Med 2004 164 1641 1649 10.1001/archinte.164.15.1641 15302634

[B9] Goldenberg DL Multidisciplinary modalities in the treatment of fibromyalgia J Clin Psychiatry 2008 69 Suppl 2 30 34 18537461

[B10] Riemsma RP Kirwan JR Taal E Rasker JJ Patient education for adults with rheumatoid arthritis Cochrane Database Syst Rev 2003 CD003688 12804484 10.1002/14651858.CD003688

[B11] Sim J Adams N Systematic review of randomized controlled trials of nonpharmacological interventions for fibromyalgia Clin J Pain 2002 18 324 336 10.1097/00002508-200209000-00008 12218504

[B12] Mease P Arnold LM Bennett R Boonen A Buskila D Carville S Fibromyalgia syndrome J Rheumatol 2007 34 1415 1425 17552068

[B13] Barlow J Wright C Sheasby J Turner A Hainsworth J Self-management approaches for people with chronic conditions: a review Patient Educ Couns 2002 48 177 187 10.1016/S0738-3991(02)00032-0 12401421

[B14] Lorig KR Mazonson PD Holman HR Evidence suggesting that health education for self-management in patients with chronic arthritis has sustained health benefits while reducing health care costs Arthritis Rheum 1993 36 439 446 10.1002/art.1780360403 8457219

[B15] Kristjansson E Tugwell PS Wilson AJ Brooks PM Driedger SM Gallois C Development of the effective musculoskeletal consumer scale J Rheumatol 2007 34 1392 1400 17552066

[B16] Riemsma RP Taal E Kirwan JR Rasker JJ Patient education programmes for adults with rheumatoid arthritis BMJ 2002 325 558 559 10.1136/bmj.325.7364.558 12228119 PMC1124093

[B17] Tugwell PS Wilson AJ Brooks PM Driedger SM Gallois C O'Connor AM Attributes and skills of an effective musculoskeletal consumer J Rheumatol 2005 32 2257 2261 16265713

[B18] Tugwell P Idzerda L Wells GA Generic quality-of-life assessment in rheumatoid arthritis Am J Manag Care 2007 13 Suppl 9 S224 S236 18095786

[B19] Lorig KR Self-management education: history, definition, outcomes, and mechanisms Ann Behav Med 2003 26 1 1 7 10.1207/S15324796ABM2601_01 12867348

[B20] Osborne RH Spinks JM Wicks IP Patient education and self-management programs in arthritis Med J Aust 2004 180 S23 S26 14984359 10.5694/j.1326-5377.2004.tb05909.x

[B21] Beaton DE Bombardier C Guillemin F Ferraz MB Guidelines for the process of cross-cultural adaptation of self-report measures Spine 2000 25 3186 3191 10.1097/00007632-200012150-00014 11124735

[B22] Finset A Steine S Haugli L Steen E Lærum E The Brief Approach/Avoidance Coping Quesstionaire: development and validation Psychology, Health & Medicine 2002 7 75 85 10.1080/13548500120101577

[B23] Stanton AL noff-Burg S Cameron CL Ellis AP Coping through emotional approach: problems of conceptualization and confounding J Pers Soc Psychol 1994 66 350 362 10.1037/0022-3514.66.2.350 8195990

[B24] Stanton AL Kirk SB Cameron CL noff-Burg S Coping through emotional approach: scale construction and validation J Pers Soc Psychol 2000 78 1150 1169 10.1037/0022-3514.78.6.1150 10870915

[B25] Zangi HA Garratt A Hagen KB Stanton AL Mowinckel P Finset A Emotion regulation in patients with rheumatic diseases: validity and responsiveness of the Emotional Approach Coping Scale (EAC) BMC Musculoskelet Disord 2009 10 107 10.1186/1471-2474-10-107 19728869 PMC2749806

[B26] Malt UF Mogstad TE Refnin IB [Goldberg's General Health Questionnaire] Tidsskr Nor Laegeforen 1989 109 1391 1394 2749623

[B27] Sampogna F Picardi A Chren MM Melchi CF Pasquini P Masini C Association between poorer quality of life and psychiatric morbidity in patients with different dermatological conditions Psychosom Med 2004 66 620 624 10.1097/01.psy.0000132869.96872.b2 15272112

[B28] Pembroke TP Rasul F Hart CL Davey SG Stansfeld SA Psychological distress and chronic obstructive pulmonary disease in the Renfrew and Paisley (MIDSPAN) study J Epidemiol Community Health 2006 60 789 792 10.1136/jech.2005.042150 16905724 PMC2566028

[B29] Benjamin S Morris S McBeth J Macfarlane GJ Silman AJ The association between chronic widespread pain and mental disorder: a population-based study Arthritis Rheum 2000 43 561 567 10.1002/1529-0131(200003)43:3<561::AID-ANR12>3.0.CO;2-O 10728749

[B30] Finset A Anke AW Hofft E Roaldsen KS Pillgram-Larsen J Stanghelle JK Cognitive performance in multiple trauma patients 3 years after injury Psychosom Med 1999 61 576 583 10443768 10.1097/00006842-199907000-00024

[B31] Goldberg D Williams P A user's guide to the General Health Questionnaire 1988 Winsor (UK), NFER-Nelson Publishing Company 1 129 Ref Type: Report

[B32] Malt UF The validity of the General Health Questionnaire in a sample of accidentally injured adults Acta Psychiatr Scand Suppl 1989 355 103 112 10.1111/j.1600-0447.1989.tb05260.x 2624128

[B33] McDowell I Psychological Well-being Measuring Health. A guide to Rating Scales and Questionnaires 2006 3 New York: Oxford University Press 206 272

[B34] Polit D Beck C Developing and Testing Self-Report Scales Nursing Research, Generating and Assessing Evidence for Nursing Practice 2008 8 Philadelphia: Lippingcott Williams & Wilkins 474 505

[B35] Andresen EM Criteria for assessing the tools of disability outcomes research Arch Phys Med Rehabil 2000 81 S15 S20 10.1053/apmr.2000.20619 11128900

[B36] Bland JM Altman DG Measuring agreement in method comparison studies Stat Methods Med Res 1999 8 135 160 10.1191/096228099673819272 10501650

[B37] de Croon EM Sluiter JK Nijssen TF Dijkmans BA Lankhorst GJ Frings-Dresen MH Predictive factors of work disability in rheumatoid arthritis: a systematic literature review Ann Rheum Dis 2004 63 1362 1367 10.1136/ard.2003.020115 15479885 PMC1754825

[B38] Kjeken I Dagfinrud H Mowinckel P Uhlig T Kvien TK Finset A Rheumatology care: Involvement in medical decisions, received information, satisfaction with care, and unmet health care needs in patients with rheumatoid arthritis and ankylosing spondylitis Arthritis Rheum 2006 55 394 401 10.1002/art.21985 16739186

[B39] Polit D Beck C Using Inferential Statistics to Test Hypothesis Nursing Research, Generating and Assessing Evidence for Nursing Practice 2008 8 Philadelphia: Lippingcott Williams & Wilkins 583 614

[B40] Cohen J Statistical power analysis for the behavioural sciences 1988 2 Hillsdale, New Jersey: Lawrance Erlbaum Associates

[B41] Anderson LW Krathwohl DR Airiasian W Cruikshank KA Mayer RE Pintrich PR A Taxonomy for Learning, teaching and assessing - A revision of Bloom's Taxonomy of educational outcomes: Complete edition 2001 Addison Wesley Longman, Inc

[B42] Specialized Health Services Act. 2-7-1999 Ref Type: Statute

